# TolC plays a crucial role in immune protection conferred by *Edwardsiella tarda* whole-cell vaccines

**DOI:** 10.1038/srep29488

**Published:** 2016-07-12

**Authors:** Chao Wang, Bo Peng, Hui Li, Xuan-xian Peng

**Affiliations:** 1 Center for Proteomics and Metabolomics, State Key Laboratory of Bio-Control, MOE Key Lab Aquat Food Safety, School of Life Sciences, Sun Yat-sen University, University City, Guangzhou 510006, People’s Republic of China; 2 Freshwater fisheries Academy of Shandong province, Jinan 250117, People’s Republic of China

## Abstract

Although vaccines developed from live organisms have better efficacy than those developed from dead organisms, the mechanisms underlying this differential efficacy remain unexplored. In this study, we combined sub-immunoproteomics with immune challenge to investigate the action of the outer membrane proteome in the immune protection conferred by four *Edwardsiella tarda* whole-cell vaccines prepared via different treatments and to identify protective immunogens that play a key role in this immune protection. Thirteen spots representing five outer membrane proteins and one cytoplasmic protein were identified, and it was found that their abundance was altered in relation with the immune protective abilities of the four vaccines. Among these proteins, TolC and OmpA were found to be the key immunogens conferring the first and second highest degrees of protection, respectively. TolC was detected in the two effective vaccines (live and inactivated-30-F). The total antiserum and anti-OmpA titers were higher for the two effective vaccines than for the two ineffective vaccines (inactivated-80-F and inactivated-100). Further evidence demonstrated that the live and inactivated-30-F vaccines demonstrated stronger abilities to induce CD8+ and CD4+ T cell differentiation than the other two evaluated vaccines. Our results indicate that the outer membrane proteome changes dramatically following different treatments, which contributes to the effectiveness of whole-cell vaccines.

Vaccines are the most effective strategy to control infectious diseases caused by pathogens^1^,^2^. The type of vaccine can be differentiated based on preparation methods, of which whole-cell vaccines were the earliest developed and are currently in wide use^3^. There are two types of whole bacterial vaccine: the live vaccine and the inactivated vaccine. Inactivated vaccines comprise several subtypes, categorized based on the method of vaccine preparation^4^. In most cases, higher immune protection is detected with live vaccines than with inactivated cells or with vaccines prepared at lower temperatures compared to higher temperatures^5^,^6^. The strong immune protection induced by live vaccines is attributed to the possibility that live vaccines may mimic natural infection, including secreted proteins, and thus naturally evoke the full immune response of the host^7^,^8^. This hypothesis partly explains the differential immune protective abilities of different types of vaccines but does not actually provide answers regarding how inactivated vaccines derived from the same cells but treated via different methods lead to the induction of differential immune protection. Given that bacterial proteins may stimulate or inhibit host immune responses^9^,^10^, we reasoned that whole-cell vaccines stimulate host immunity by placing all surface proteins in contact with the host immune system rather than a single surface protein; thus, the resulting immunity derives from the immune responses stimulated by all of the surface proteins, regardless of whether they stimulate or inhibit protective immunity. Elucidation of the mechanisms involved in immune stimulation by whole cell vaccines may enhance our understanding of how a host responds to a whole cell vaccine and facilitate the identification of effective protective immunogens within the proteome.

Recent advances in biotechnology now allow for a deep understanding of vaccine mechanisms in the context of vaccine development, particularly the use of transcriptomics and proteomics methodologies^11^,^12^,^13^. Immunoproteomics based on the combination of 2-DE proteomics and Western blotting is an efficient tool for the identification of immunogens^12^,^13^. Immunogen identification is particularly important for a whole-cell vaccine because it contains many proteins. However, studies regarding the mechanisms underlying immune protection in response to whole-cell vaccines are not available.

*Edwardsiella tarda* is an intracellular pathogen that causes severe economic loss in fish. In some situations, vaccines are more economical and effective than antibiotics; antibiotics are efficient for managing bacterial infections but can result in the production of antibiotic-resistant bacteria^14^,^15^. Recently, whole-cell *E. tarda* vaccines have been investigated, and studies have indicated that formalin-killed cells are ineffective in protecting against *E. tarda* infection, whereas a live attenuated vaccine strategy is more effective^16^,^17^. However, the mechanisms underlying this differential protection are largely unknown. Here, we show that when *E. tarda* cells underwent differential treatment, the outer membrane proteome was significantly altered, including TolC, an immunogen that is key to mounting an effective immune response. These altered proteomes were found to be related to differential immune protective ability.

## Results

### Differential Immune Protection Conferred by Four Types of Bacterial Whole-Cell Vaccines

To investigate the mechanism underlying the notion that live vaccines confer better protection efficacy, four methods were used to prepare vaccines, leading to the generation of live, inactivated-30-F, inactivated-80-F and inactivated-100 vaccines. Mice were challenged with *E. tarda* EIB202 post-immunization with these vaccines. Different vaccines exhibited significantly different levels of protection. Protective rates were 50%, 35%, 20% and 10% for the live, inactivated-30-F, inactivated-80-F, and inactivated-100 vaccines, respectively, while control mice experienced a cumulative death rate of 100% (Table 1). There were significant differences in the protective rates between the live or inactivated-30-F and inactivated-80-F or inactivated-100 vaccines. These data indicate that protective ability is determined by the method of vaccine preparation, and live bacteria demonstrated the strongest protective ability.

### Identification of Immunogens using Immunoproteomics

**Figure 1:**
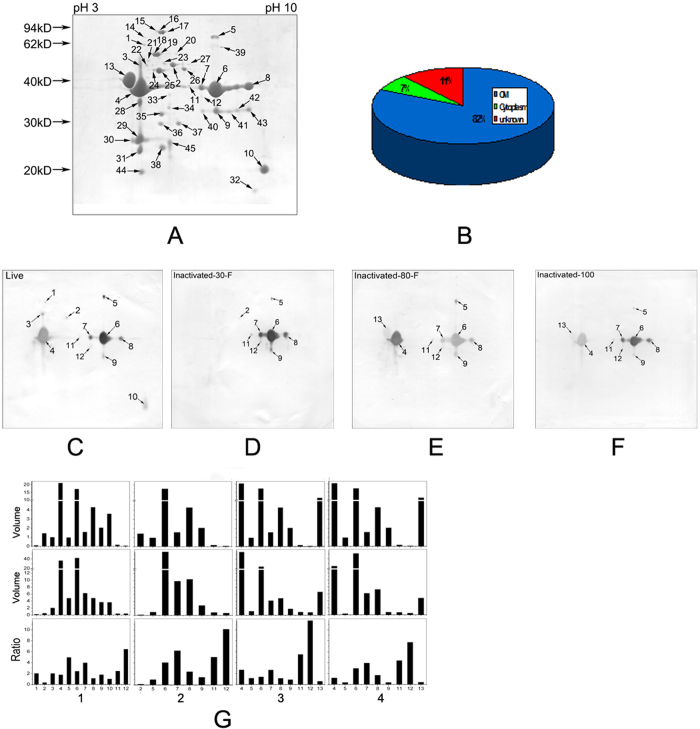
Profile of *E. tarda* outer membrane proteins and immunoproteomics. (**A**) 2-DE map for *E. tarda* outer membrane proteins. (**B**) Pie chart indicating the locations of the identified proteins in the 2-DE gel. (**C–F**) Immunoproteomics using antisera prepared following immunization with live bacteria (**C**), inactivated-30-F (**D**), inactivated-80-F (**E**) and inactivated-100 (**F**) bacteria. (**G**) Volume and ratio of the spots from 2-DE gels and 2-DE Western blotting. The volume of spots obtained from staining with Coomassie brilliant blue in the 2-DE gel (upper), staining with DAB for immunoproteomics (middle) and the ratio between the upper and middle (lower). Numbers 1–13 represent spots. 1, GroEL, 2, TolC, 3 and 4, OmpF2, 5–9, OmpA, 10, ETAE_2675, 11 and 12, OmpA, 13, ETAE_1826. 1–4 indicate different antisera prepared from mice immunized with live bacteria (1), inactivated-30-F (2), inactivated-80-F (3) and inactivated-100 (4) vaccines.

Based on the above results, we postulated that high temperature might denature the outer membrane proteins, thereby changing their immunogenicity to initiate an immune response. 2-DE-based proteomics and immunoproteomics were used to investigate the mouse immune response to outer membrane proteins in the four vaccines. First, we established a 2-DE profile and identified 45 protein spots (Fig. 1A and Table S1), of which 37 (82%) were outer membrane proteins, 5 (11%) were cytoplasmic proteins and 3 (7%) were proteins of unknown location (Fig. 1B). The abundance of these proteins was greatly altered among vaccine groups. OmpF2 (spot 4), OmpA (spot 6) and ETAE_1826 (spot 13) were the three proteins with the highest abundance (Fig. 1A). Several proteins had more than one spot, e.g., OmpA had 12 spots, indicating post-translational modification^18^.

Then, we applied immunoproteomics to characterize the protein spots recognized by mouse anti-sera (Fig. 1C–F), which were prepared separately from five mice each vaccinated with the four vaccines. In total, 13 spots representing 6 proteins were identified, including GroEL (spot 1), TolC (spot 2), OmpF2 (spots 3, 4), ETAE_2675 (spot 10), ETAE_1826 (spot 13) and OmpA (spots 5, 6, 7, 8, 9, 11, 12). All of the positive proteins with the exception of GroEL were outer membrane proteins. The sera produced by the different vaccines reacted differentially with these spots. Specifically, 12, 8, 9 and 9 spots representing 5, 2, 3 and 3 proteins were detected in the live (Fig. 1C), inactivated-30-F (Fig. 1D), inactivated-80-F (Fig. 1E) and inactivated-100 (Fig. 1F) vaccines, respectively. These proteins were GroEL, TolC, OmpF2, OmpA, and ETAE_2675 in live bacteria, TolC and OmpA in inactivated-30-F bacteria, and OmpF2, OmpA and ETAE_1826 in both inactivated-80-F and inactivated-100 bacteria. These results indicate that differential antibody responses were detected among the four vaccines.

Interestingly, TolC was recognized only by the two anti-sera generated against live and inactivated-30-F vaccines. OmpA was detected in all four vaccines. However, we failed to detect OmpF2 in inactivated-30-F following several repeated attempts, indicating the loss of OmpF2 during preparation. Therefore, TolC might be a key protein for the generation of protective immunity. In addition, in consistent with our previous reports, the present study also indicated that the volume percentage of a protein spot in 2-DE was not consistent with that of the staining intensity in 2-DE Western blotting^12^,^13^. Equally importantly, this study further revealed that different protein spots for the same protein showed differential staining intensities when reacting with the same antiserum. For example, several OmpA spots with differential abundance did not produce similar ratios in their ability to bind to the same antibody, suggesting the effects exerted by polyclonal antibodies and OmpA modifications on binding. Generally, stronger staining and higher ratios of spots 5–9 (OmpA) were detected in the live and inactivated-30-F vaccines than in the inactivated-80-F and inactivated-100 vaccines (Fig. 1G). In addition, ETAE_2675 is an outer membrane protein that was recognized only in the sera generated against the inactivated-80-F and inactivated-100 vaccines.

### Protective ability of outer membrane immunogens

**Figure 2:**
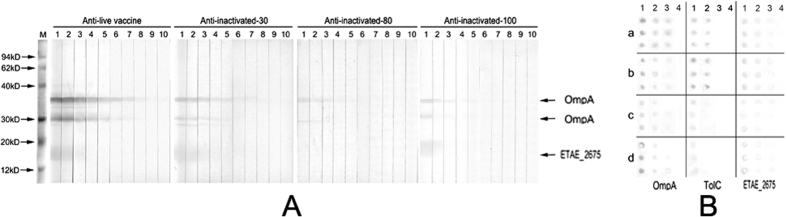
Titers of the four antisera. (**A**) Total antisera titers. M, marker. 1–10: antisera dilutions at 1:100, 1:200, 1:400, 1:800, 1:1600, 1:3200, 1:6400, 1:12800, 1:25600 and 1:51200. Three clearly positive bands are shown by the arrows. (**B**) Titers of antisera raised against the single purified proteins OmpA, TolC and ETAE_2675. 1–4: antisera dilutions at 1:100, 1:400, 1:1600 and 1:6400. a–d, Antisera prepared following immunization by the live (a), inactivated-30-F (b), inactivated-80-F (c) and inactivated-100 (d) vaccines.

We further investigated the protective abilities of TolC, OmpA and ETAE_2675 using active immunization in a mouse model. The results from the active immunization showed that the protection rates of TolC, OmpA and ETAE_2675 were 60.8%, 45.1% and 2%, respectively (Table 2), indicating that TolC and OmpA are good protective immunogens. OmpA is a conserved outer membrane protein in Gram-negative bacteria and an effective immunogen against bacterial infections^12^,^13^,^19^. Although the same dose of bacteria (10^7^ cells/mouse) was used to immunize with all four vaccines, the resulting intensities were dramatically different in Western blots. The evaluation of intensity resulted in the ranking of the vaccines: live, inactivated-30-F, inactivated-80-F and inactivated-100; the titers produced by these vaccines were 1:25600, 1:6400, 1:1600 and 1:400, respectively (Fig. 2A). Therefore, the titers appeared to be associated with the immune protection conferred, although antibody titer is not equivalent to immune protection^20^.

The anti-sera titers for the outer membrane proteins TolC, OmpA and ETAE_2675 were determined by Dot-ELISA. Consistent with the above results, stronger staining and higher titers of the three proteins were detected in the anti-sera against the live or inactivated-30-F vaccines than the other two vaccines (Fig. 2B). Interestingly, the lower abundance of OmpA in inactivated-30-F could induce higher titers of anti-serum, including antibodies against OmpA, than the other two inactivated vaccines, suggesting that outer membrane proteins in addition to OmpA play a role in mounting the immune response, including regulation of the generation of high titers of anti-OmpA. These results indicate that the live and inactivated-30-F vaccines produce higher titers of antibodies against immunogenic outer membrane proteins, which is related to the presence of other outer membrane proteins that react with the host.

### Effects of different vaccine preparations on the abundance of outer membrane proteins

**Figure 3:**
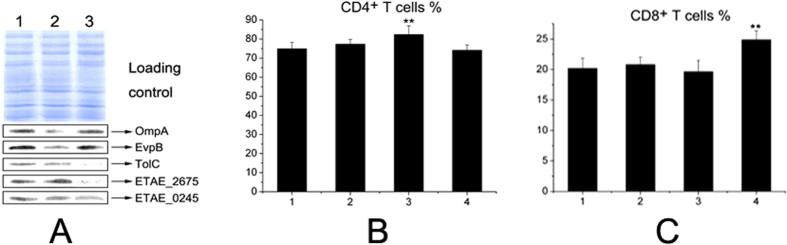
Western blotting analysis to determine the abundance of outer membrane proteins (**A**) and flow cytometry analysis to determine the percentages of CD4+ (**B**) and CD8+ (**C**) T cells. (**A**) Three EIB202 vaccines. 1, Live vaccine; 2, inactivated-30-F vaccine; 3, inactivated-100 vaccine. (**B,C**) CD3+ T cells from mice immunized with the three vaccines. 1, PBS control; 2, inactivated-100 vaccine; 3, inactivated-30-F vaccine; 4, live vaccine. **p < 0.01 using Student’s T test.

Based on the above results, we reasoned that the abundance of outer membrane proteins might be altered following different treatments. To demonstrate this, we used Western blotting to investigate whether the outer membrane proteins OmpA, EvpB, TolC, ETAE_2675 and ETAE_0245 showed changes in their expression levels in the live, inactivated-30-F and inactivated-100 vaccines. The inactivated-80-F vaccine was not included because it was not lysed by ultrasonication despite several repeated attempts. The results showed that TolC and ETAE_2675 were lost in inactivated-100. OmpA and EvpB were lower in the inactivated-30-F vaccine than in the other vaccines (Fig. 3A). These results indicate that the immune protective abilities of whole-cell vaccines result from different vaccine preparation methods, and these abilities can be attributed to the differential abundance of outer membrane proteins.

Next, we used a flow cytometry assay to analyze T cell differentiation. Using fluorescently labeled antibodies, CD4+ and CD8+ T cells were counted within the CD3+ T cell subpopulation. The percentage of CD4+ T cells was significantly higher in mice immunized with the inactivated-30-F vaccine than in mice immunized with the other three vaccines, while the percentage of CD8+ T cells was significantly higher in mice immunized with the live vaccine than mice in the other three groups (Fig. 3B,C). The results shows that the inactivated-30-F vaccine and live vaccine induced CD4+ and CD8+ T cell differentiation to trigger humoral immunity and cellular immunity, respectively.

### Effects of different antibiotic-stressed vaccines on their protective abilities in fish and mouse models

**Figure 4:**
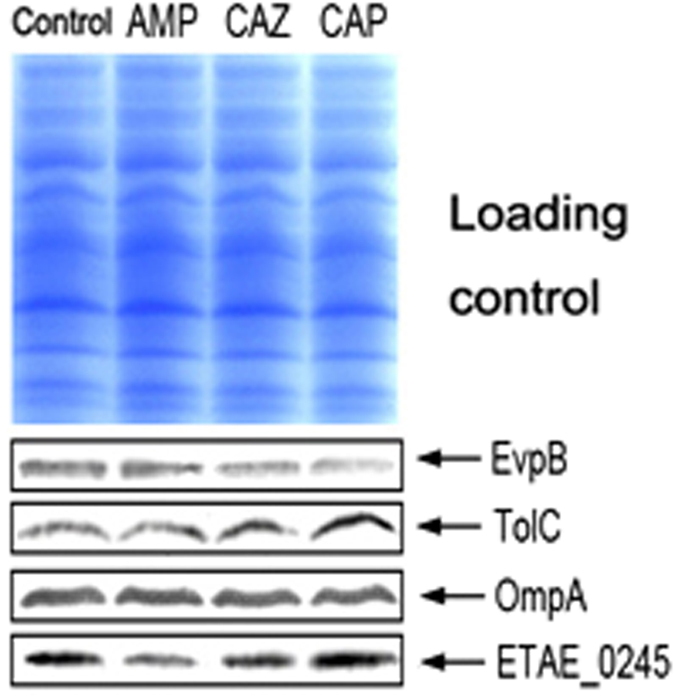
Differential regulation of outer membrane proteins in antibiotic-stressed vaccines. LTB4 vaccines prepared by exposure to the antibiotics AMP (ampicillin), CAP (chloramphenicol) and CAZ (ceftazidime).

To further support the conclusion that the immune protective abilities of whole-cell vaccines are related to the differential abundance of outer membrane proteins, we prepared three other vaccines from the same LTB4 cells, but in this case the cells were stressed by exposing them separately to ampicillin, chloramphenicol and ceftazidime for 1 h. It is possible that antibiotic stress might result in changes in the abundance of outer membrane proteins irrespective of gene mutations^21^,^22^. LTB4 rather than EIB202 cells were used for the investigation to ensure that our findings were not limited to EIB202 cells. Our pre-test showed that LTB4 possessed similar outer membrane proteins to EIB202. Out of the four proteins detected by Western blotting, three proteins (OmpA was the exception) showed significant differences among the three vaccines, among which TolC had the highest abundance in the vaccine prepared by exposure to chloramphenicol (Fig. 4). Further investigation of immune protection found that a significantly higher protective ability was detected for the chloramphenicol-stressed vaccine than the control vaccine in fish and mouse models, whereas there was no significant difference between the other two vaccines and the control (Table 3). When fish were challenged by *E. tarda* EIB202, some of the animals died, mostly 24–48 h post challenge. Moribund fish exhibited typical symptoms of *E. tarda* infection, including skin inflammation with depigmentation and hemorrhage on the surface of the tail. Moreover, the infection was validated by detection of *E. tarda* 16S rRNA in livers of the dying fish and mice (Supplementary Fig. 1). The results further support our conclusions described above. Meanwhile, these findings suggest that we may assess vaccine quality by detecting vaccine-efficient proteins such as TolC and anti-OmpA, as detailed in this study.

## Discussion

Although differential immune protection conferred by different vaccines that were derived from the same bacterium but prepared via different treatments has been observed^4^,^23^, the mechanisms underlying this phenomenon are still unknown. In the present study, we applied sub-immunoproteomics to investigate the relationship between differential outer membrane proteomes and immune protection by whole-cell bacterial vaccines prepared by different methods, as well as to identify key immunogens. The outer membrane proteome was selected because outer membrane proteins, which are located at the outermost area of bacterial cells and are the first contact between a bacterium and host cells and the environment^24^,^25^, are ideal targets for vaccines^12^,^13^,^26^,^27^,^28^,^29^,^30^.

Our results indicate that the immune protection abilities of vaccines are associated with outer membrane proteome, and treatment conditions such as temperature can alter the outer membrane proteome, leading to altered immune protection ability. The conditions used to inactivate live bacteria, such as formalin or temperature, may exert potential effects on protein structures, which results in the loss of antigenic epitopes and abundance and in turn affects host stimulation. In the present study, a high abundance of outer membrane proteins was more beneficial for antibody production. Thus, it is important to avoid decreases in the abundance of outer membrane protective immunogens during vaccine preparation. In contrast, the present study demonstrated lower OmpA but higher anti-OmpA levels in inactivated-30-F compared to the inactivated-80-F and inactivated-100 vaccines. This finding indicates that the ability of a protein to induce high antibody titers is related not only to the identity of the protein but also to the actions of other proteins. Significantly differential protein abundance and components were detected among the four vaccines, and OmpF2 was lost in the inactivated-30-F vaccine in the present study. These results also suggest that other proteins in addition to protective immunogens in the vaccine also play a role in inducing high antibody titers, which could be another reason why differential immune protection was detected among the four *E. tarda* vaccines produced via different treatments. Correspondingly, TolC abundance and anti-OmpA may be of importance to identify the effectiveness of these vaccines. The detection of the abundance of key proteins during vaccine preparation could be an alternative method to predict vaccine efficiency.

Our results indicate that the immune protective abilities of whole cell vaccines are attributable to the actions of all outer membrane proteins that interact with the host immune system rather than only those proteins that activate immune protection. Therefore, the differential abundances of outer membrane proteins contribute to the differential immune protection abilities of the vaccines. Our results highlight a method to enhance vaccine efficiency through the regulation of ratios among vaccine components.

Interestingly, our results show that the inactivated-30-F vaccine and live vaccine were able to induce CD4+ and CD8+ T cell differentiation. CD4 is a co-receptor that assists the T cell receptor (TCR) in communicating with an antigen-presenting cell through direct interaction with MHC class II molecules on the surface of the antigen-presenting cell. The interaction further mediates downstream signal transduction via tyrosine phosphorylation, leading to T cell activation to trigger humoral immunity. Cytotoxic T cells with CD8 surface proteins are called CD8+ T cells. The extracellular IgV-like domain of CD8-α interacts with the α3 portion of the Class I MHC molecule, keeping the T cell receptor of the cytotoxic T cell and the target cell bound closely together during antigen-specific activation and thereby triggering cellular immunity^31^,^32^.

Additionally, out of the two protective immunogens identified in the present study, the immune protective antigen *E. tarda* OmpA was reported previously^30^. Furthermore, the immune protective role of OmpA has been revealed in other bacterial species. This may be due to the conservative role of OmpA in Gram-negative bacteria^12^,^13^,^30^. However, information regarding to the role of *E. tarda* TolC is not available, although immune protection conferred by *Salmonella paratyphi* A TolC has been reported^33^. Our results obtained from active immunization indicate that a higher level of protection could be detected in mice immunized with TolC compared to OmpA. Thus, the present study introduces an efficient novel immunogen to control infections caused by *E. tarda.*

## Methods

### Ethics statement

All work was conducted in strict accordance with the recommendations in the Guide for the Care and Use of Laboratory Animals of the National Institutes of Health. The protocol was approved by the Institutional Animal Care and Use Committee of Sun Yat-sen University (Animal Welfare Assurance Number: I6).

### Bacterial Strains and Animals

The bacterial strains *E. tarda* EIB202 and LTB4 used in the present study were obtained from Professor Yuanxin Zhang, East China University of Science and Technology, and Professor Xiaohua Zhang, Ocean University of China, respectively. The two bacterial strains have been reported in many studies from our lab and other labs^18^,^34^. The complete genome sequence of EIB202 was published in 2009^18^. The strains were grown in tryptic soy broth (TSB) at 30 °C and harvested at 1.0 of OD_600_. SPF Kunming mice were obtained from the Animal Center of Sun Yat-sen University and fed sterile water and a dry pellet diet. Tilapias were purchased from the Guangzhou Tilapias Breeding Base; the fish were 5 ± 0.5 cm in length and 1.8 ± 0.2 g in body weight and were acclimated in stock tanks (80 × 75 × 90 cm). The animals were fed commercial pelleted feed twice per day. After acclimating for one week, the fish were demonstrated to be free of *E. tarda* species through microbiological and PCR detections. The animals were then randomly divided into several groups for immunization.

### Vaccine preparation and active immunization

For vaccine preparation and immunization, EIB202 cells with an OD of 1.0 were harvested by centrifugation at 4,000 g for 15 min and washed three times with saline solution. The resulting cells were suspended in sterile saline solution as live *E. tarda* vaccine or then incubated at different temperatures (30 °C plus 0.5% formalin, 80 °C plus 0.5% formalin or 100 °C) to produce the inactivated-30-F, inactivated-80-F and inactivated-100 vaccines, respectively. Plate counting was performed to examine bacterial sterility in the three inactivated vaccines. The inactivated cells (10^7^ cells) and living bacteria (10^7^ CFU) were administered to the mice by intraperitoneal injection. After two injections at an interval of 7 days, the mice were challenged by EIB202 at 5 × 10^8^ CFU and observed daily for 15 days. For bacterial challenge post immunization, mice were injected with recombinant OmpA, EvpB, TolC, ETAE_2675 or ETAE_0245 with Freund’s complete adjuvant and boosted with Freund’s incomplete adjuvant at an interval of 7 days with 100 μg per mouse per injection. The control group was injected with phosphate buffered saline (PBS) containing equal amounts of Freund’s complete or incomplete adjuvant. At 7 days after the booster immunization, the mice were challenged with EIB202 at 5 × 10^8^ CFU and observed daily for 15 days. Three dying mice in each group were randomly selected to isolate liver for use in PCR analysis of 16S rRNA.

### *E. tarda* LTB4 vaccines prepared by exposure to antibiotics

LTB4 cells at an OD_600_ of 1.0 of were collected and washed three times with saline solution. The cells were separately exposed at 8-fold minimum inhibitory concentrations (MICs) of the antibiotics (100 μg/mL ampicillin, 8 μg/mL chloramphenicol and 1 μg /mL ceftazidime) at 30 °C for 1 h. The resulting bacteria were collected and divided into two parts. One was used for Western blotting to detect the abundance of outer membrane proteins and another was inactivated with 0.5% formalin for 90 min at 30 °C for mouse immunization as described above. After immunization two times, the mice were challenged with *E. tarda* EIB202 at 4.5 × 10^8^ CFU. Meanwhile, tilapias were immunized with 8.0 × 10^3^ CFU of LTB4 cells twice at an interval of ten days and then were challenged with *E. tarda* EIB202 at 8 × 10^4^ CFU. These animals were observed daily for 15 days. The experiment was repeated three times for Western blotting and twice for challenge post immunization. Three dying fish in each group were randomly selected to isolate liver for use in PCR analysis of 16S rRNA.

### Detection of 16S rRNA

Standard PCR was used to amplify 16S rRNA gene of mouse blood before infection and liver after infection, and fish liver before and after infection. A pairs of primers for gene 16 sRNA were designed with the sense primer 5′-AGAGTTTGATCCTGGCTCA-3′ and the antisense primer 5′-GGTTACCTTGTTACGACTT-3′. The PCR started with 4 min at 94 °C, followed by 33 cycles with 30 s at 94 °C, 30 s at 57 °C, 90 s at 72 °C, and a final extension for 10 min at 72 °C. The resulting PCR products were sequenced by BGI, Shenzhen, Guangdong and then compared with the parent *E. tarda* strain for validation of the infectious pathogen.

### Isolation of outer membrane proteins

Outer membrane proteins were separated with lauryl sarcosinate as previously described^35^. In brief, bacterial cells were harvested by centrifugation at 4,000 g for 15 min at 4 °C. The resulting cells were washed in 40 mL of sterile saline solution (0.15 M NaCl) three times and then resuspended in 5 mL of 50 mM Tris-Cl pH 7.2. These cells were disrupted by intermittent ultrasonic treatment. Unbroken cells and cellular debris were removed by centrifugation at 5,000 g for 20 min. Supernatants were collected and further centrifuged at 100,000 g for 1 h at 4 °C. The precipitate was dissolved in 2% (W/V) sodium lauryl sarcosinate at room temperature for 30 min and ultra-centrifuged again. The collected pellets were resuspended in 50 mM Tris–Cl and stored at −80 °C. The concentrations of these proteins in the final preparation were determined using the Bradford method.

### 2-DE-based proteomics and immunoproteomics

2-DE based proteomics and immunoproteomics were performed as described previously^12^,^13^,^21^. In brief, before 2-DE was performed, the samples were treated with TCA-acetone. After rehydration overnight, 200 μg of outer membrane proteins in 200 μL of rehydration buffer were loaded on linear 11-cm pH 3–10 immobilized pH gradient strips (IPG strips, BioRad, USA). The IPG strips were focused in an IPGphor at 20 °C and 60 kVh using the Multiphor II system (Amersham). After reduction and alkylation by DTT and IAA, respectively, the IPG strips were transferred for second-dimension electrophoresis using 12% acrylamide gels. The gels were stained with Coomassie Blue-R250 and scanned in an AGFA white-light scanner. The gel patterns were matched to each other by visual comparison using the 2-D software Melanie 5.0. Differentially expressed proteins were excised from 2-D gels, digested with trypsin and applied for MALDI-TOF/MS analysis (Reflex III MALDI-TOF system, Bruker). MS peaks were selected between 800 and 3,000 Daltons and filtered with a signal-to-noise ratio greater than 15 and to exclude masses derived from trypsin autolysis. Proteins with low confidence were further identified using MALDI TOF/TOF. For MS/MS spectra, the 5 most abundant precursor ions per sample were selected for subsequent fragmentation, and 1000–1200 Da laser shots were accumulated per precursor ion. The criterion for precursor selection was a minimum S/N of 50. All MALDI analyses were performed with a fuzzy logic feedback control system (Reflex III MALDI-TOF system, Bruker) equipped with delayed ion extraction. Both the MS and MS/MS data were interpreted and processed using Flexanalysis 3.0 (Bruker Daltonics), and then the obtained MS and MS/MS spectra per spot were combined and submitted to the MASCOT search engine (V2.3, Matrix Science, London, U.K.) by Biotools 3.1 (Bruker Daltonics) and searched with the following parameters: NCBI in SwissProt (http://www.matrixscience.com), one missed cleavage site, carbamidomethyl as a fixed modification of cysteine and oxidation of methionine as a variable modification, MS tolerance of 100 ppm, and MS/MS tolerance of 0.6 Da. Known contaminant ions (keratin) were excluded. A 95% confidence level threshold was used for MASCOT protein scores. Proteins were identified by a MASCOT score higher than 78 for peptide mass fingerprinting and an ion score higher than 49 for MS/MS analysis. Other criteria for the identification included at least 8% sequence coverage for MS and at least two peptides for MS/MS. Each sample had 3 biological repeats. The sub-cellular protein locations were positioned with the program PSORTb version 2.0.4 (http://www.psort.org/psortb/). For immunoproteomics analysis, proteins in the gels were transferred onto NC membranes, incubated with primary and secondary antibodies and reacted with DAB substrate until the optimal color appeared. The primary antibodies were prepared by immunizing mice with the live (live), inactivated at 30 °C for 24 h plus 0.5% formalin (inactivated-30-F), at 80 °C for 90 min plus 0.5% formalin (inactivated-80-F) and at 100 °C for 1 h (inactivated-100) vaccines. The secondary antibody, rabbit anti-mouse-IgG-HRP, was commercially obtained from Boson Biotech. Co., Ltd, Xiamen, China. The experiment was repeated three times.

### Gene cloning and protein purification

A pair of primers for the *tolC* gene were designed based on the EIB202 genomic sequence (sense, 5′ GGG**GGATCC**ATGAAGAAACTGCTCCC 3′; antisense, 5′ CCC**AAGCTT**TTAGCGTACGCCGCCGCC 3′), while primer sequences for the four genes *ompA*, *evpB*, ETAE_0245 and ETAE_2675 were available in our previous report^19^. Standard PCR and molecular biology protocols were utilized to amplify the genes using EIB202 genomic DNA as a template. The PCR fragment was directionally cloned into the plasmid pET-32a and then expressed in *E. coli* BL21. The recombinant plasmid was detected by restriction enzyme analysis and sequencing. Sequencing was carried out by BGI, Shenzhen, China. The recombinant proteins were purified by affinity chromatography on Ni-NTA Super flow resin (Qiagen) according to the manufacturer’s instructions. The concentration of proteins was determined by the Bradford method. The purified recombinant proteins, OmpA, EvpB, TolC, ETAE_2675, and ETAE_0245, were used for antiserum preparation (Guangzhou Chengxue Biotech. Corp. China).

### Western blotting and Dot-ELISA

To detect protein abundance, the prepared vaccines were used for the extraction of outer membrane proteins as described above. These outer membrane proteins were separated by SDS-PAGE, transferred to NC membranes and then incubated with antiserum prepared from the live vaccine. To detect antiserum titers, outer membrane proteins of the live vaccine were used as antigens and were recognized by antisera separately against the live vaccine, inactivated-30-F, inactivated-80-F and inactivated-100 bacteria. Western blotting was carried out with a routine procedure as described above for immunoproteomics. The experiment was repeated three times.

For dot ELISA, 0.5 μg of recombinant OmpA, TolC and ETAE_2675 were separately dotted onto an NC membrane. After blocking with 5% skim milk, the membranes were recognized separately by four mouse antisera raised against the four vaccines at dilutions of 1:100, 1:400, 1:1600 and 1:6400. After incubating with a secondary antibody, rabbit anti-mouse IgG-HRP, the membranes were reacted with DAB substrate until spots appeared. The experiment was repeated three times.

### Flow cytometry analyses of T cells

Mice were immunized using live, inactivated-30-F and inactivated-100 vaccines and boosted at an interval of one week for tested groups, and PBS was used as a control. After the second immunization, heparin anticoagulant blood was collected for flow cytometry analysis. Two milliliters of red cell lysis solution was added and incubated for 10 min. Cell debris was removed by centrifugation at 1,500 rpm for 5 min. FITC-conjugated anti-CD3 mAb, PE-conjugated anti-CD4 mAb and APC-conjugated anti-CD8 mAb (BD Biosciences) were added into each 100-μL fresh sample and incubated for 15 min in the dark. After washing with 2 mL of PBS, the cells were resuspended in 400 μL of PBS and analyzed by flow cytometry analysis. The experiment was repeated three times.

### Statistical analysis

Chi-square tests and Student’s T tests were performed with SPSS software version 11.5 (SPSS Inc.) to determine statistical significance. Significant differences were considered present at *P < 0.05 and **P < 0.01.

## Additional Information

**How to cite this article**: Wang, C. *et al*. TolC plays a crucial role in immune protection conferred by *Edwardsiella tarda* whole-cell vaccines. *Sci. Rep.* **6**, 29488; doi: 10.1038/srep29488 (2016).

## Supplementary Material

Supplementary Information

## Figures and Tables

**Table 1:** Active immunization protection of the four bacterial vaccines in mice.

Vaccine	No. of mice	ADR %	RPS %
Control	20	100%	0
Live	20	50%	50% **
Inactivated-30-F	20	65%	35% **
Inactivated-80-F	20	80%	20%
Inactivated-100	20	90%	10%

ADR: accumulating death rate; RPS: relative percent survival, for which the calculation was RPS = 1 − (ADR of vaccinated group/ADR of control group) ×100. **P°0.01 (compared with the control group) using the Chi-square test.

**Table 2:** Active immune protection of the three proteins in mice.

Vaccine	No.	ADR %	RPS %
PBS	20	85.0	0
OmpA	15	46.7	45.1 **
TolC	15	33.3	60.8 **
ETAE_2675	15	83.3	2

ADR: accumulating death rate; RPS: relative percent survival, **P < 0.01 using the Chi-square test.

**Table 3:** Active immune protection induced by antibiotic-stressed vaccines in mouse and tilapia models.

Vaccine	No.	Mouse ADR %	RPS %	Tilapia ADR %	RPS %
PBS	42	59.5	0.0	59.5	0.0
AMP-stressed	30	70.0	−17.6	66.7	−17.6
CAZ-stressed	30	66.7	−12.0	60.0	−5.82
CAP-stressed	30	33.3	44.0 *	26.7	52.9 *

Note: *E. tarda* LTB4 cells were treated with PBS (phosphate buffer saline), AMP (ampicillin), CAZ (ceftazidime), or CAP (chloramphenicol) and were prepared as control, AMP-stressed, CAZ-stressed and CAP-stressed vaccines, respectively. Then, animals were immunized with these vaccines and challenged by EIB202. ADR: accumulating death rate; RPS: relative percent survival, *P < 0.05 using the Chi-square test.
