# Supplementary material for: TolC plays a crucial role in immune protection conferred by Edwardsiella tarda whole-cell vaccines
Source: Sci Rep. 2016 Jul 12;6:29488. doi: 10.1038/srep29488 (PMC4942608; doi:10.1038/srep29488)
Supplement: Supplementary Information [file srep29488-s1.doc]

**TolC plays a crucial role in immune protection of** ***Edwardsiella tarda* whole-cell vaccines**

Chao Wang, Bo Peng, Hui Li, Xuan-xian Peng

**Supplementary Tab. 1** **Identiﬁcation of *E. tarda* Outer Membrane Proteins Using MS and Subcellular Location**

| Spot NO. | Accession name | NCBI accession No. | locus_tag | Protein description | Subcellular location | No. of peptides matched | Cover % | Mr/pI | Sequence |  | NCBI score |
| --- | --- | --- | --- | --- | --- | --- | --- | --- | --- | --- | --- |
| 1 | D0ZAK2_EDWTE | gi|269137672 | ETAE_0314 | chaperonin GroEL (HSP60 family) | Cytoplasm | 18 | 33 | 57517/4.82 |  |  | 129 |
| 2 | D0Z9U8_EDWTE | gi|269137549 | ETAE_0191 | outer membrane channel protein | OM | 17 | 49 | 51409/6.64 |  |  | 183 |
| 3 | D0ZFQ0_EDWTE | gi|269138593 | ETAE_1239 | putative outer membrane porin F protein | M/OM | 18 | 66 | 40058/5.03 |  |  | 124 |
| 4 | D0ZFQ0_EDWTE | gi|269138593 | ETAE_1239 | putative outer membrane porin F protein | M/OM | 18 | 71 | 40058/5.03 |  |  | 161 |
| 5 | D0ZFS8_EDWTE | gi|269138621 | ETAE_1267 | outer membrane protein A | M/OM | 3 | - | 38034/7.66 | R.AALINCLAPDRR.V K.LSYPLMDDLDVYTR.L R.SETGARPDNTMLSLGVVYR.F |  | 211^*^ |
| 6 | D0ZFS8_EDWTE | gi|269138621 | ETAE_1267 | outer membrane protein A | M/OM | 1 | - | 38034/7.66 | K.IVQVIGAVVDVEFPQDAVPR.V |  | 114^*^ |
| 7 | D0ZFS8_EDWTE | gi|269138621 | ETAE_1267 | outer membrane protein A | M/OM | 5 | - | 38034/7.66 | K.DGSVVVLGYTDR.I  R.AALINCLAPDRR.V  K.LSYPLMDDLDVYTR.L  R.SETGARPDNTMLSLGVVYR.F  R.FGQDEVAAPAPIPAPAPAPVVETKR.F |  | 356^*^ |
| 8 | D0ZFS8_EDWTE | gi|269138621 | ETAE_1267 | outer membrane protein A | M/OM | 3 | - | 38034/7.66 | K.LSYPLMDDLDVYTR.L  R.SETGARPDNTMLSLGVVYR.F  R.SETGARPDNTMLSLGVVYR.F + Oxidation (M) |  | 74^*^ |
| 9 | D0ZFS8_EDWTE | gi|269138621 | ETAE_1267 | outer membrane protein A | M/OM | 25 | 69 | 38034/7.66 |  |  | 245 |
| 10 | D0ZCH1_EDWTE | gi|269140020 | ETAE_2675 | virulence-related outer membrane protein | M/OM | 2 | - | 20476/8.89 | K.AFSESTGLEYSR.G  R.DVWVGGYEEGSLQQTR.S |  | 143^*^ |
| 11 | D0ZFS8_EDWTE | gi|269138621 | ETAE_1267 | outer membrane protein A | M/OM | 3 | - | 38034/7.66 | K.LSYPLMDDLDVYTR.L  R.SETGARPDNTMLSLGVVYR.F  R.FGQDEVAAPAPIPAPAPAPVVETKR.F |  | 329^*^ |
| 12 | D0ZFS8_EDWTE | gi|269138621 | ETAE_1267 | outer membrane protein A | M/OM | 3 | - | 38034/7.66 | R.AALINCLAPDRR.V  K.LSYPLMDDLDVYTR.L  R.SETGARPDNTMLSLGVVYR.F |  | 216^*^ |
| 13 | D0Z894_EDWTE | gi|269139173 | ETAE_1826 | putative outer membrane protein (porin) | M/OM | 27 | 74 | 42331/4.68 |  |  | 280 |
| 14 | D0Z9N4_EDWTE | gi|269139520 | ETAE_2175 | 30S ribosomal protein S1 | ribosome | 17 | 27 | 61174/4.89 |  |  | 134 |
| 15 | D0ZC49_EDWTE | gi|269137961 | ETAE_0603 | organic solvent tolerance protein | M/OM | 50 | 52 | 90359/5.45 |  |  | 422 |
| 16 | D0ZC49_EDWTE | gi|269137961 | ETAE_0603 | organic solvent tolerance protein | M/OM | 53 | 58 | 90359/5.45 |  |  | 435 |
| 17 | D0ZC49_EDWTE | gi|269137961 | ETAE_0603 | organic solvent tolerance protein | M/OM | 44 | 46 | 90359/5.45 |  |  | 248 |
| 18 | D0ZB31_EDWTE | gi|269139775 | ETAE_2430 | type VI secretion system protein EvpB | unknown | 33 | 57 | 54499/5.16 |  |  | 302 |
| 19 | D0ZB32_EDWTE | gi|269139775 | ETAE_2430 | type VI secretion system protein EvpB | unknown | 2 | - | 54499/5.17 | R.YIALALPHVLGR.L  K.AVAYLKPHFQLEGLTASLR.L |  | 114^*^ |
| 20 | D0ZHG9_EDWTE | gi|269140872 | ETAE_3531 | F0F1-type ATP synthase, alpha subunit | M/IM/OM | 27 | 52 | 55302/5.59 |  |  | 281 |
| 21 | D0Z9U8_EDWTE | gi|269137549 | ETAE_0191 | outer membrane channel protein | OM | 20 | 57 | 51409/6.64 |  |  | 155 |
| 22 | D0Z9I9_EDWTE | gi|269139475 | ETAE_2130 | putative flagellin associated protein | OM | 15 | 46 | 43733/5.01 |  |  | 117 |
| 23 | D0Z9U8_EDWTE | gi|269137549 | ETAE_0191 | outer membrane channel protein | OM | 19 | 50 | 51409/6.64 |  |  | 189 |
| 24 | D0Z9X1_EDWTE | gi|269137572 | ETAE_0214 | maltoporin | M/OM | 3 | - | 47297/5.38 | R.NTANDVYDIR.L  R.NTESGGSYSYFVNQSNEK.S  K.NNWNTDQDGIQVNTQPGDIR.A |  | 301^*^ |
| 25 | D0Z9X1_EDWTE | gi|269137572 | ETAE_0214 | maltoporin | M/OM | 13 | 33 | 47297/5.38 |  |  | 99 |
| 26 | D0ZB52_EDWTE | gi|269139796 | ETAE_2451 | long-chain fatty acid transport protein | OM | 20 | 48 | 47578/6.45 |  |  | 204 |
| 27 | D0Z9U8_EDWTE | gi|269137549 | ETAE_0191 | outer membrane channel protein | OM | 20 | 57 | 51409/6.64 |  |  | 155 |
| 28 | D0ZFQ0_EDWTE | gi|269138593 | ETAE_1239 | putative outer membrane porin F protein | M/OM | 20 | 66 | 40058/5.03 |  |  | 129 |
| 29 | D0ZA02_EDWTE | gi|269137603 | ETAE_0245 | hypothetical protein ETAE_0245 | OM | 15 | 74 | 22398/5.25 |  |  | 159 |
| 30 | D0ZA02_EDWTE | gi|269137603 | ETAE_0245 | hypothetical protein ETAE_0245 | OM | 13 | 56 | 22398/5.25 |  |  | 128 |
| 31 | D0ZA02_EDWTE | gi|269137603 | ETAE_0245 | hypothetical protein ETAE_0245 | OM | 8 | 58 | 22398/5.25 |  |  | 85 |
| 32 | D0Z7Z3_EDWTE | gi|269139072 | ETAE_1723 | hypothetical protein ETAE_1723 | unknown | 8 | 47 | 12695/7.67 |  |  | 112 |
| 33 | D0Z7I1_EDWTE | gi|269138910 | ETAE_1559 | PTS system, mannose-specific IIAB component | Cytoplasm | 17 | 50 | 35031/5.40 |  |  | 165 |
| 34 | D0Z9A3_EDWTE | gi|269137488 | ETAE_0130 | outer membrane phospholipase A | M | 15 | 38 | 33603/5.97 |  |  | 104 |
| 35 | D0ZDY9_EDWTE | gi|269140278 | ETAE_2935 | nucleoside-specific channel-forming protein Tsx | OM | 11 | 32 | 32786/5.62 |  |  | 116 |
| 36 | D0Z9X1_EDWTE | gi|269138621 | ETAE_1267 | outer membrane protein A | OM | 18 | 54 | 38034/7.66 |  |  | 135 |
| 37 | D0ZH45_EDWTE | gi|269138831 | ETAE_1480 | MltA-interacting MipA family protein | unknown | 12 | 39 | 31771/7.10 |  |  | 114 |
| 38 | Q6EE21_EDWTA | gi|40287636 |  | EvpA(Protein of unknown function ) | unknown | 2 | - | 19379/5.29 | K.GVHPHLSFR.T  K.FVSIDRDNFNDVIK.G |  | 54^*^ |
| 39 | D0ZHA5_EDWTE | gi|269140808 | ETAE_3467 | vitamin B12/cobalamin outer membrane transporter | M/OM | 23 | 38 | 67868/7.77 |  |  | 241 |
| 40 | D0Z9X1_EDWTE | gi|269138621 | ETAE_1267 | outer membrane protein A | OM | 3 | - | 38034/7.66 | K.LSYPLMDDLDVYTR.L  K.LGWSHFISNSFEDMGTTK.S  R.SETGARPDNTMLSLGVVYR.F |  | 278^*^ |
| 41 | D0Z9X1_EDWTE | gi|269138621 | ETAE_1267 | outer membrane protein A | OM | 21 | 64 | 38034/7.66 |  |  | 227 |
| 42 | D0Z9X1_EDWTE | gi|269138621 | ETAE_1267 | outer membrane protein A | OM | 3 | - | 38034/7.66 | K.DGSVVVLGYTDR.I  K.LSYPLMDDLDVYTR.L  R.SETGARPDNTMLSLGVVYR.F |  | 235^*^ |
| 43 | D0Z9X1_EDWTE | gi|269138621 | ETAE_1267 | outer membrane protein A | OM | 21 | 59 | 38034/7.66 |  |  | 180 |
| 44 | D0ZAV3_EDWTE | gi|269139697 | ETAE_2352 | hypothetical protein ETAE_2352 | unknown | 2 | - | 18864/5.45 | K.DYTEATGGVR.W R.WNVMRPLSVDVGYR.Y |  | 119^*^ |
| 45 | D0ZH93_EDWTE | gi|269138879 | ETAE_1528 | outer membrane protein W | OM | 4 | - | 23320/6.17 | -.HEAGEFFMR.A K.LGGAQQHDSVR.L R.LDPWVFMFSAGYR.F K.DSWGAAGQVGVDYLINR.D K.FRPYVGAGINYTTFFDNGFNDHGK.E R.ATGDIATVHHLPPTLMAQWYFGDASSK.F |  | 689^*^ |

^*^ MS/MS results

**Sequencing primer: 27F**


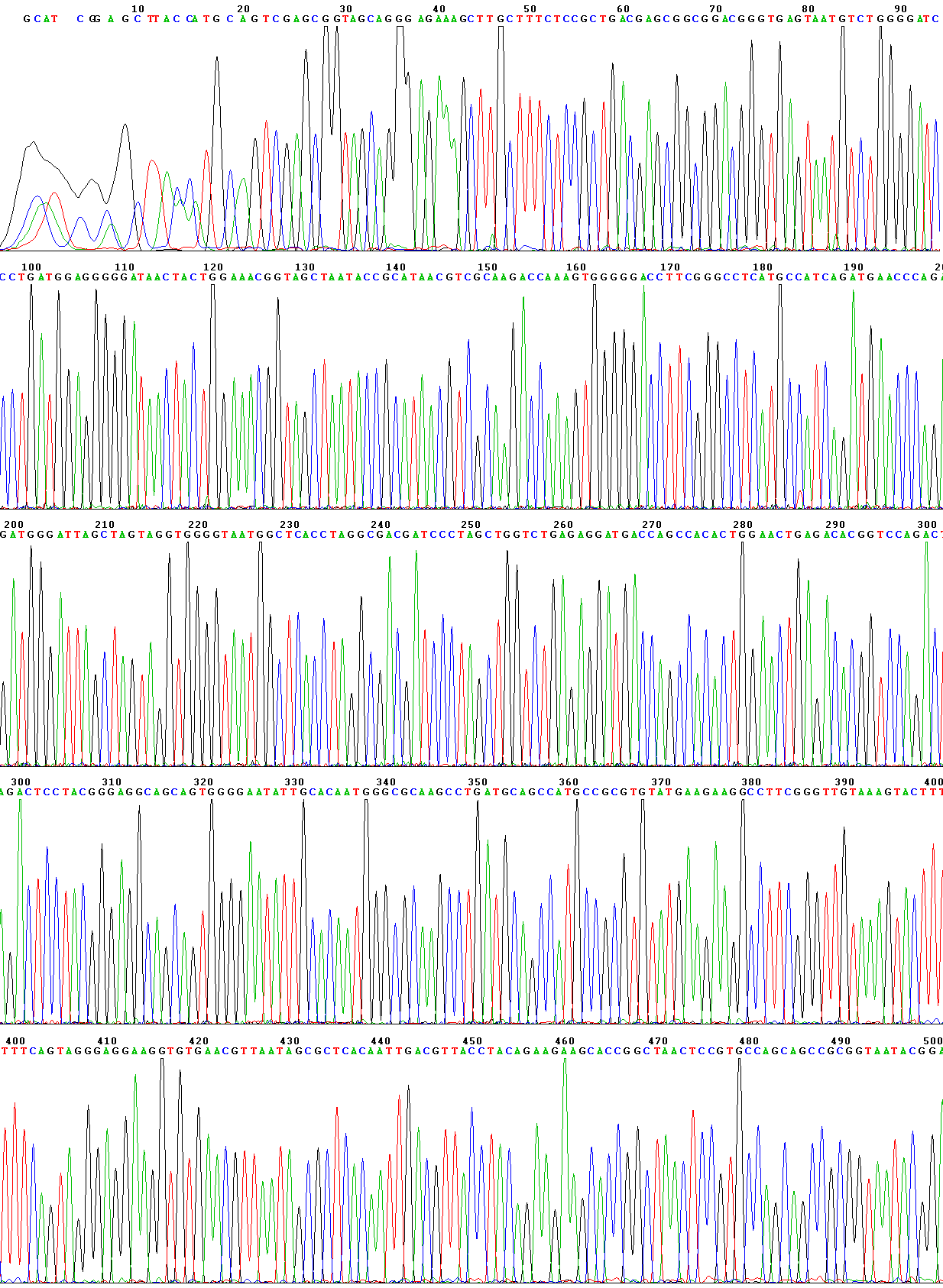


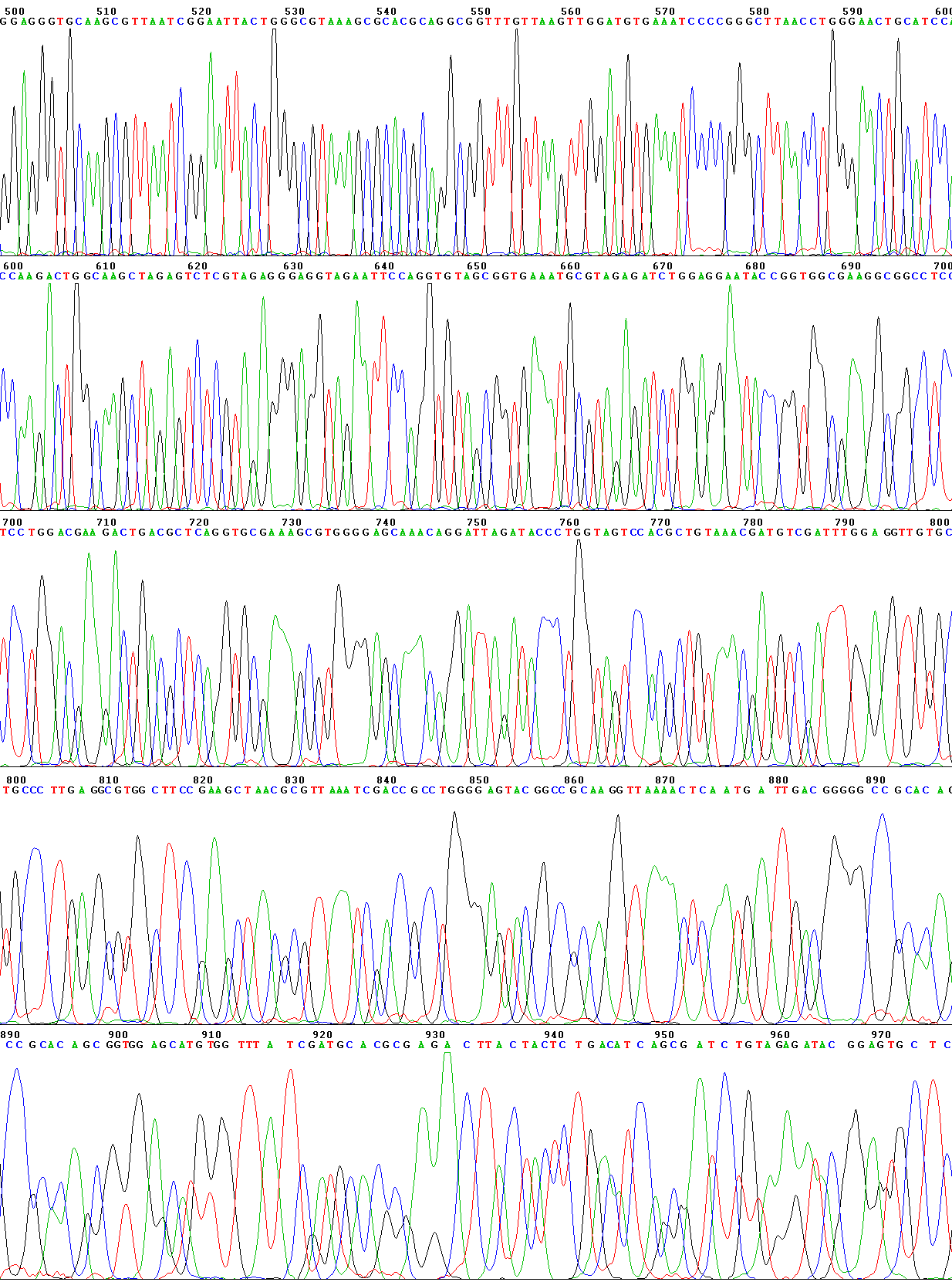


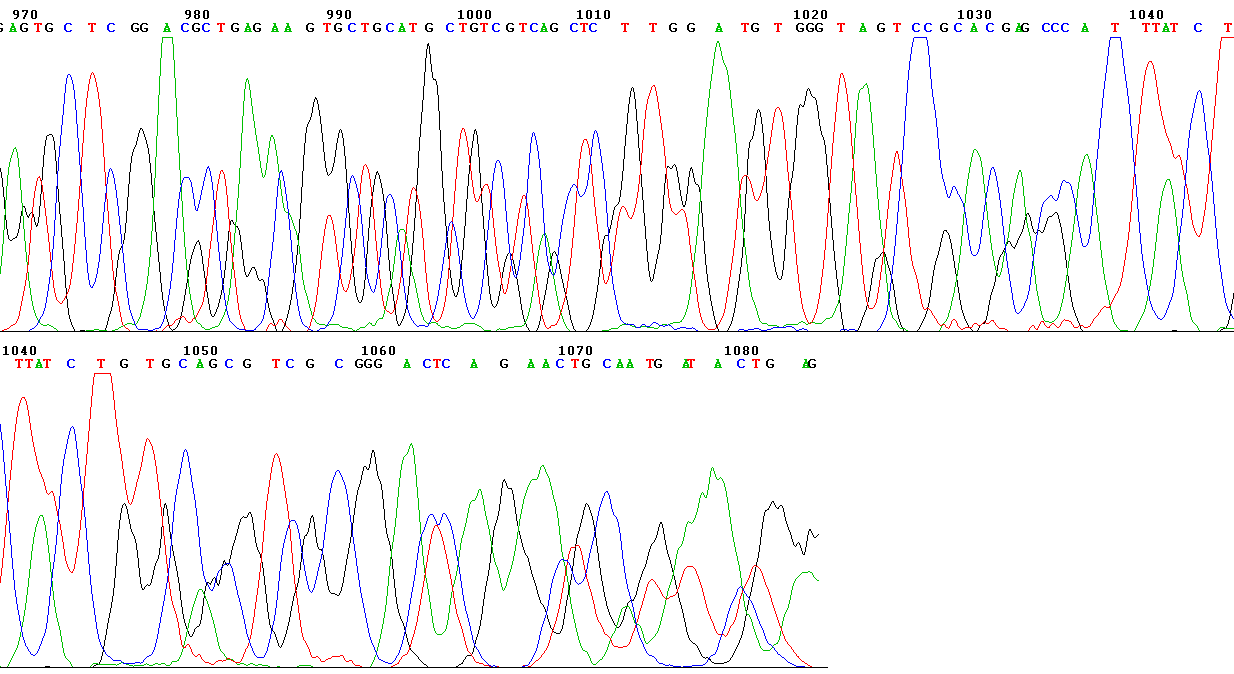


**Sequencing primer: 1492R**


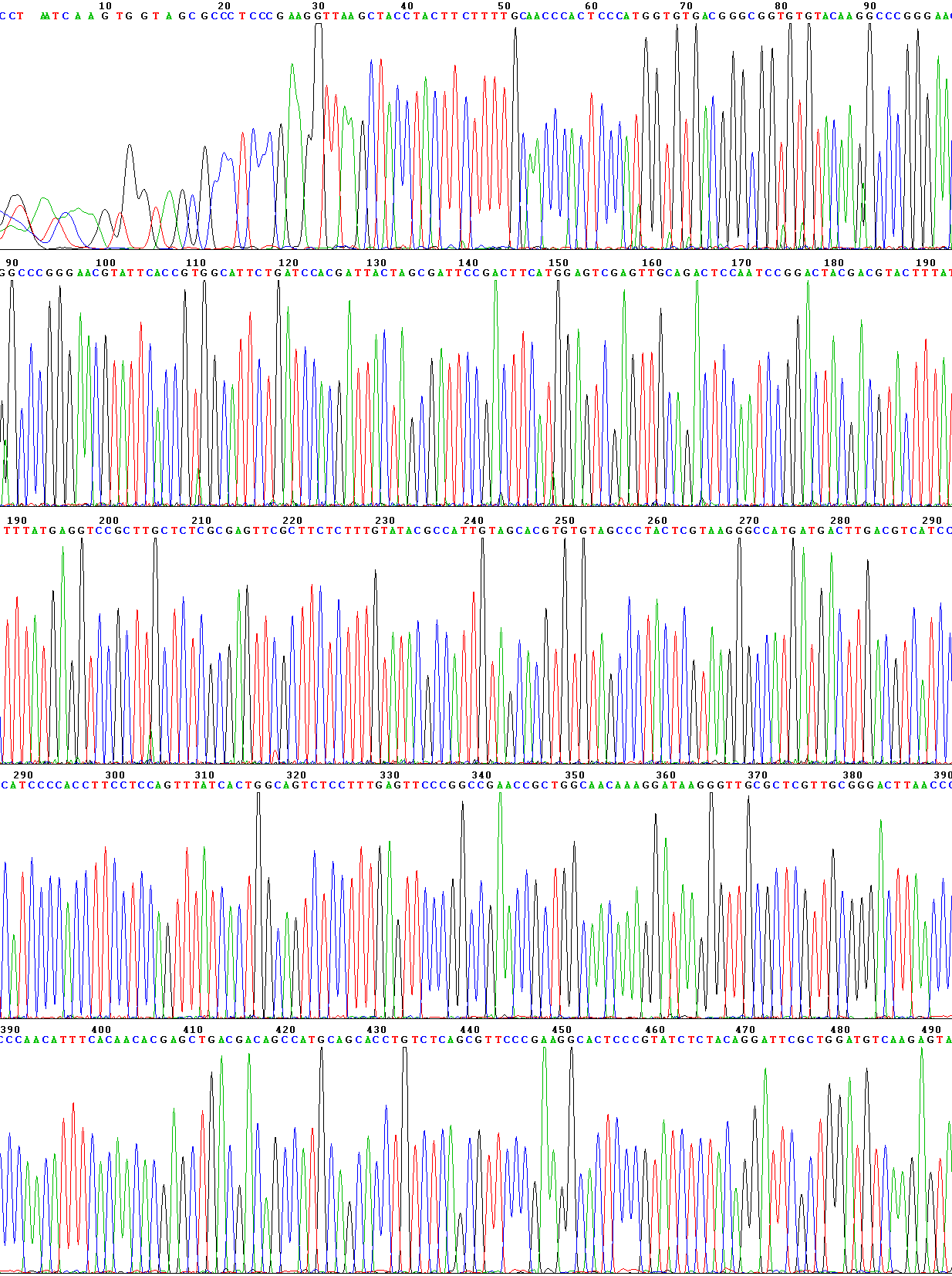


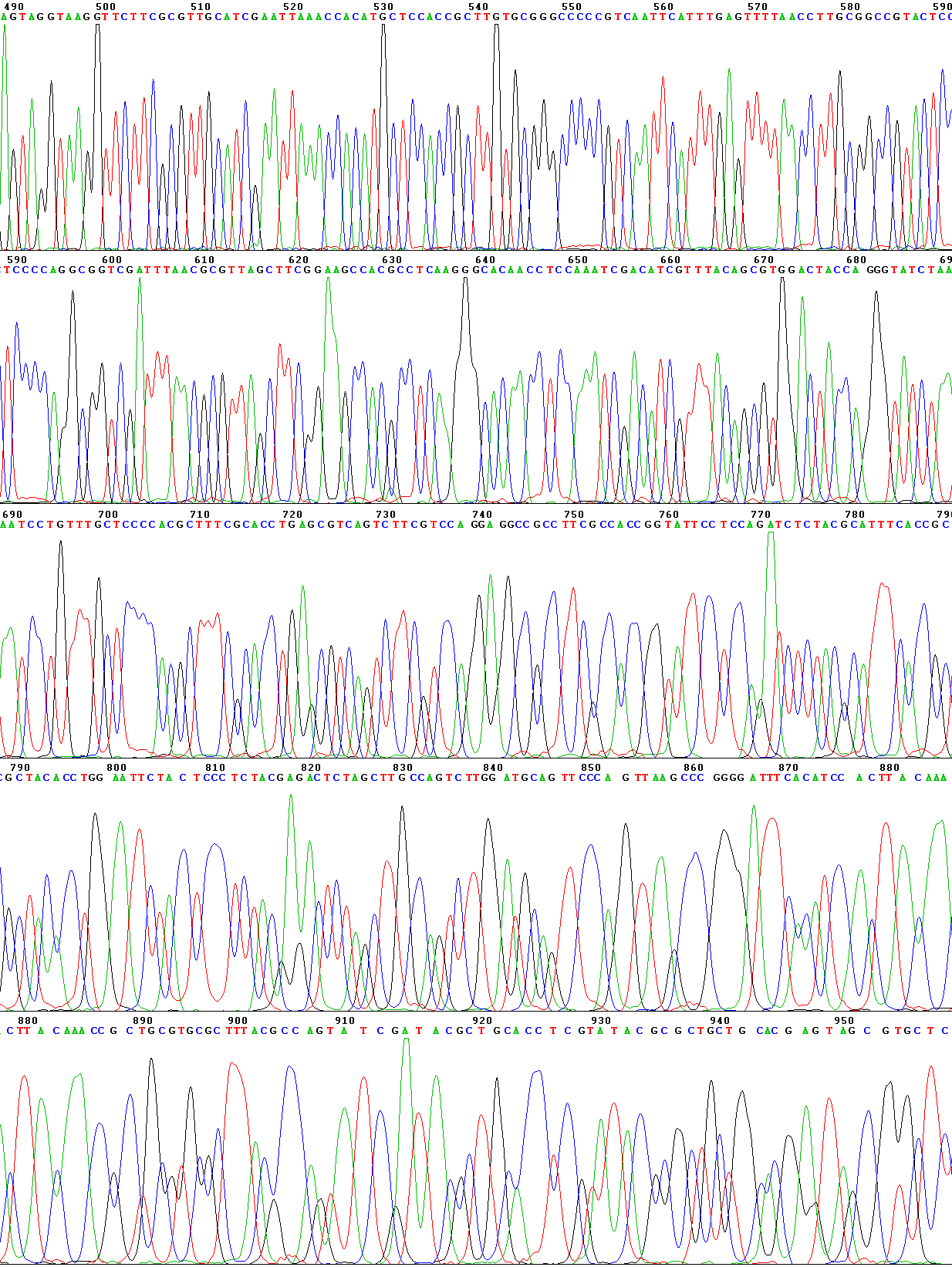


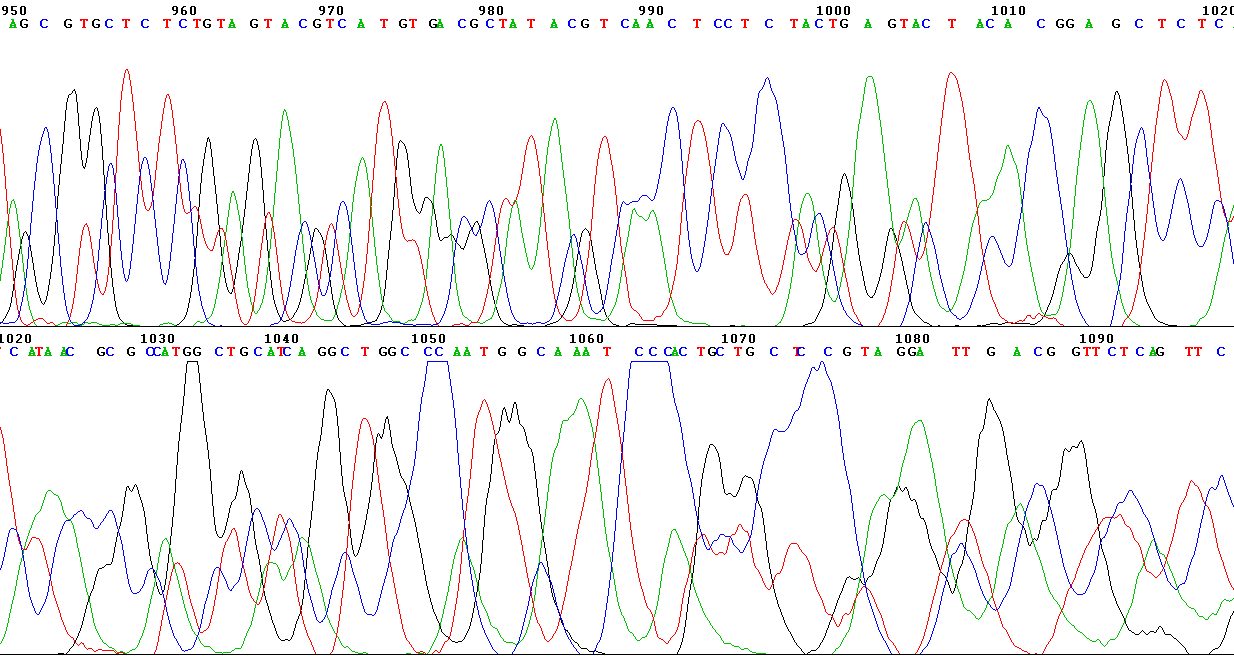


**Supplementary Figure 1 Sequencing diagram of 16S rRNA of EIB202**
